# ARTP/NTG Compound Mutagenesis Improved the Spinosad Production and the Insecticidal Virulence of *Saccharopolyspora Spinosa*

**DOI:** 10.3390/ijms252212308

**Published:** 2024-11-16

**Authors:** Zirong Zhu, Wangqiong Chen, Li Cao, Ziyuan Xia, Jie Rang, Shengbiao Hu, Liqiu Xia

**Affiliations:** State Key Laboratory of Developmental Biology of Freshwater Fish, Hunan Provincial Key Laboratory of Microbial Molecular Biology, College of Life Science, Hunan Normal University, Changsha 410081, China; 201820141158@hunnu.edu.cn (Z.Z.); 202320142772@hunnu.edu.cn (W.C.); 201920142230@hunnu.edu.cn (L.C.); 201910140184@hunnu.edu.cn (Z.X.); rang0214@hunnu.edu.cn (J.R.); shengbiaohu@hunnu.edu.cn (S.H.)

**Keywords:** *Saccharopolyspora spinosa*, ARTP/NTG mutagenesis, spinosad, biotechnology

## Abstract

Spinosad is an efficient and broad-spectrum environmentally friendly biopesticide, but its low yield in wild-type *Saccharopolyspora spinosa* limits its further application. ARTP/NTG compound mutagenesis was used in this study to improve the spinosad titer of *S. spinosa* and obtain a high-yield mutant—NT24. Compared with the wild-type strain, the fermentation cycle of NT24 was shortened by 2 days and its maximum titer of spinosad reached 858.3 ± 27.7 mg/L, which is 5.12 times more than for the same-period titer of the wild-type strain. In addition, RT-qPCR, resequencing, and targeted metabolomics showed that the upregulation of the key differential genes *accD6*, *fadD*, *sdhB*, *oadA*, and *gntZ* caused increased metabolic flux in the tricarboxylic acid cycle and pentose phosphate pathway, suggesting that the accumulation of pyruvate and short-chain acyl-CoA was the primary cause of spinosad accumulation in NT24. This study demonstrates the effectiveness of ARTP mutagenesis in *S. spinosa*, and provides new insights for the mechanism of spinosad biosynthesis and metabolic engineering in *S. spinosa*.

## 1. Introduction

*Saccharopolyspora spinosa* (*S. spinosa*), a Gram-positive filamentous bacterium, is a rare actinomycete and the source of a variety of secondary metabolites with bioactivities, such as macrolides [1,2,3]. *S. spinosa* was originally isolated from soil samples from the Caribbean islands in 1982, and is capable of producing macrolide secondary metabolites with high insecticidal activity after aerobic fermentation [4,5]. These bioactive substances have been defined as spinosad [6]. A macrolide compound, spinosad is composed of a twelve-membered lactone ring, a 5-6-5 cis-trans-cis tricyclic ring, and two glycosyl groups [7]. Due to the different positions of acyl modification on the glycosyl group, spinosad has a variety of homologues in structure [8]. At least twenty-five types of spinosyn were isolated from a fermentation broth of *S. spinosa*, in which the main components were spinosyn A and spinosyn D [9]. The mixture of spinosyn A and spinosyn D was called spinosad [10], which has highly specific and insecticidal activity toward a variety of pests, e.g., Lepidoptera [11], Diptera [12], and Thysanoptera [13], as well as toxicity toward Coleoptera and Hymenoptera [14]. Spinosad mainly acts on the insect nervous system, especially the acetylcholine receptor and γ-aminobutyric acid (GABA) receptor [15]. After binding to these receptors, the nerve conduction of the target pest will be disturbed, thus causing persistent muscle contraction and nerve overexcitation and ultimately leading to paralysis and death of the target pest [16,17]. In addition, compared with traditional pesticides, spinosad leaves little environmental residue [18,19]; therefore, it has been widely used in food storage and crop pest control [20].

However, it is difficult to screen novel *S. spinosa* strains by conventional soil separation methods, and the spinosad production of the wild-type *S. spinosa* strains obtained from the natural environment is often too low to meet the needs of large-scale industrial production. Therefore, various strategies have been adopted to increase the production capacity of wild-type *S. spinosa* to enable it to adapt to industrial production in recent years [21], such as metabolic engineering [22], transcriptomics-based genetic modification [23], medium optimization [4], and random mutation [24]. Although various gene modification methods are the first choice for improving the performance of strains, in many cases, random mutation breeding is still the most effective way to change the genes and characteristics of the microbiome [25].

The *S. spinosa* genome contains high G + C content and has a large number of biosynthetic gene clusters. Using traditional genetic modification on it faces challenges such as difficulty in operation, long cycle, and unpredictable results, and the cost of trial and error is extremely high [26,27]. From a commercial standpoint, there are many limitations to the application of genetically modified products, and the strains obtained from random mutagenesis are always classified as non-genetic modifications [28]. Thus, artificial mutagenesis methods, such as physical mutagenesis, chemical mutagenesis, and physical–chemical compound mutagenesis, are often employed to accelerate the evolution of strains to obtain strains with improved performance. For physical mutagenesis, ARTP (atmospheric and room temperature plasma) mutagenesis is an emerging mutagenesis technology [29]. In the process of ARTP mutagenesis, the excited particles in plasma jets act on the strains and induce damage to the cell wall, cell membrane, and DNA or RNA, leading to changes in cell membrane permeability and the activity of various intracellular proteins, which may trigger the initiation of SOS repair (the source of mutations and new phenotypes) [30,31,32].

Compared with traditional physical mutagenesis, such as ultraviolet irradiation and gamma rays, ARTP uses a non-invasive method to mutate the strain, which is safer and can cause a higher degree of DNA damage [28,32]. In addition, ARTP mutagenesis is driven by RF power, which can be produced under normal pressure and room temperature without expensive vacuum systems or extreme temperature. The mutation distance between the plasma trigger and samples, as well as the intensity and duration of plasma jets, can be adjusted, rendering it applicable to various strains [33,34]. At present, ARTP mutagenesis has been applied to the breeding of a variety of bacteria [35,36,37].

In this study, we first employed ARTP/NTG compound mutagenesis to improve the spinosad production of *S. spinosa*. To further reveal the high-yield mechanism of spinosad in *S. spinosa*, transcription-level verification combined with resequencing and target metabolomic analysis was conducted. This study not only aimed to increase the yield of spinosad, but also to provide a presumptive mechanism of spinosad biosynthesis promotion in *S. spinosa*.

## 2. Results

### 2.1. ARTP/NTG Compound Mutagenesis Improved the Titer of Spinosad in S. spinosa

**Figure 1:**
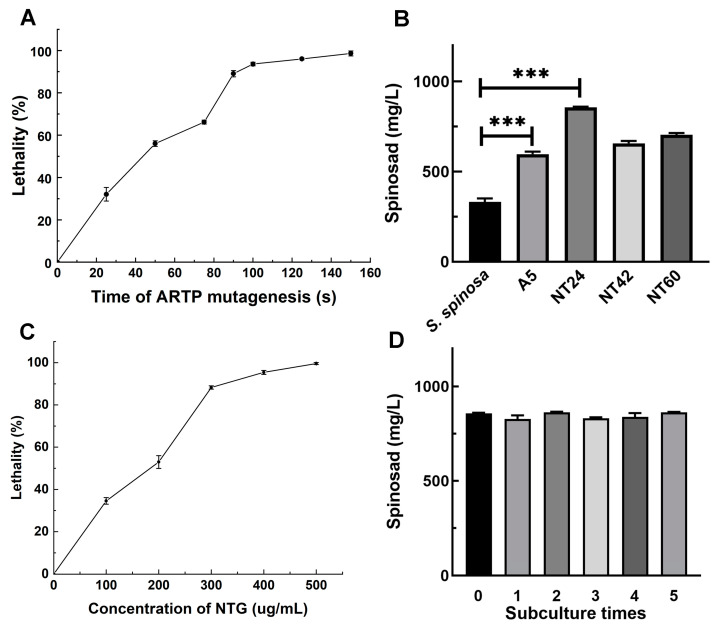
ARTP/NTG compound mutagenesis in *S. spinosa*. (**A**) The lethality rates of *S. spinosa* under different durations of ARTP treatment. (**B**) Spinosad titer of high-yield mutants of *S. spinosa* from ARTP/NTG compound mutagenesis. (**C**) The lethality rates of *S. spinosa* under different concentrations of NTG for 1 h. (**D**) Subculture of NT24 and spinosad titer of each passage. Error bars show standard deviations. Univariate variance, *** *p* < 0.001.

To determine the optimal mutation time of ARTP mutagenesis, the lethality rates of wild-type *S. spinosa* (WT) were assessed under exposure times of 0–150 s (0, 25, 50, 75, 90, 100, 125, and 150 s) (Figure 1A). As shown in Figure 1, a dose–response relationship was observed between the lethality rate of WT and ARTP treatment duration. After 90 s and 100 s exposure under ARTP, the lethality rate reached 89% and 93.6%, respectively. Consequently, 179 mutants obtained from 90 s and 100 s ARTP mutagenesis were subjected to batch fermentation for yield determination, with mutant A5 exhibiting the greatest increase in spinosad production, yielding a titer of 597.4 ± 14.5 mg/L, 1.8 times that of the WT strain (317.5 ± 12.7 mg/L) (Figure 1B).

Thus, A5 was selected as the initial strain of NTG mutagenesis. The lethality rates of A5 were determined under different NTG concentrations (0, 100, 200, 300, 400, and 500 µg/mL) to identify the optimal concentration for NTG mutagenesis (Figure 1C). The results show that 300 µg/mL NTG mutagenesis results in a lethality rate of 88.3%. After 1 h of mutagenesis with 300 µg/mL NTG, three mutants (NT24, NT42 and NT60) exhibiting improved spinosad titer were screened. Among them, the spinosad titer of mutant NT24 was the highest (858.3 ± 27.7 mg/L), which is 2.58 and 1.43 times higher than those of the WT strain and mutant A5, respectively (Figure 1B). The fermentation products were collected for mass spectrometry identification, and the mass spectrometry (MS) results revealed the [M + -H] + ions at m/z = 732.4 (Figure S1). Additionally, mutant NT24 was confirmed to exhibit stable spinosad production after five subcultures (Figure 1D).

### 2.2. Biological Activity Assay of NT24

**Figure 2:**
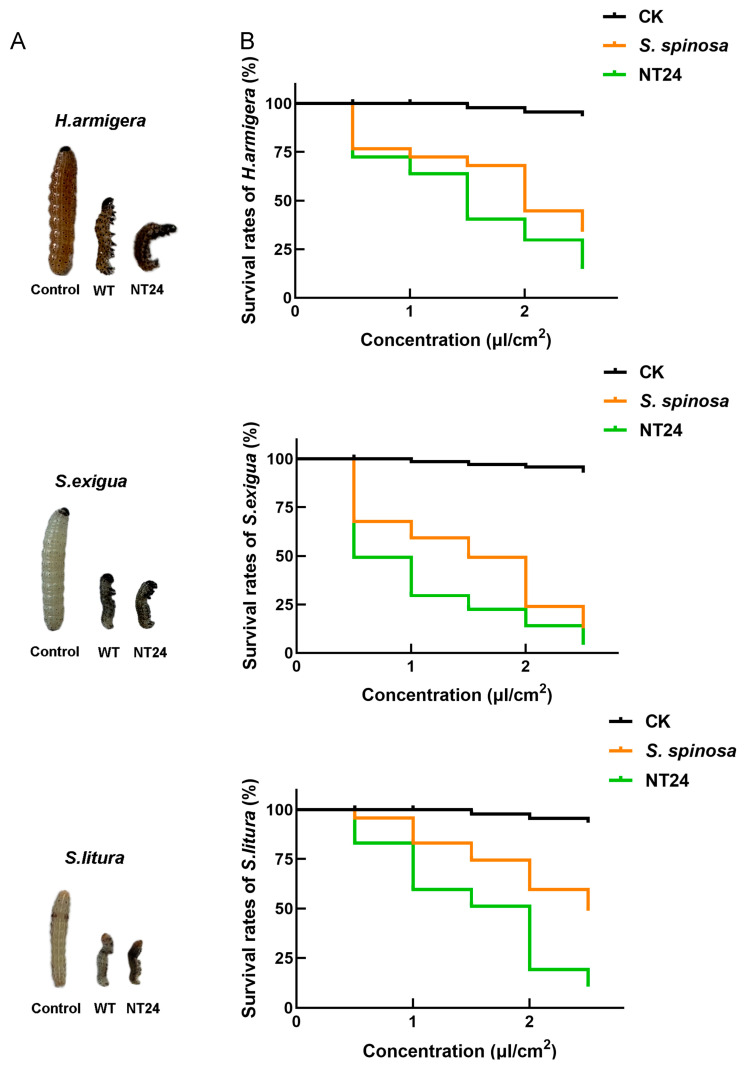
Biological activity comparison of WT and NT24. (**A**) Toxic symptoms of *H. armigera*, *S. exigua* and *S. litura* after treated with the fermentation broth (0.5 μL/cm^2^) of WT and NT24 for 48 h. (**B**) The survival rates of *H. armigera*, *S. exigua* and *S. litura* after being treated with different fermentation broths of WT and NT24 for 48 h.

Insecticidal activity experiments on three Lepidoptera pests including *H. armigera*, *S. exigua* and *S. litura* were conducted to assess the difference in the yield and virulence of spinosads between WT and NT24. Compared with the WT strain, NT24 caused higher mortality rates and exhibited stronger insecticidal virulence. Second-stage larvae of *H.armigera*, *S.exigua* and *S.litura* treated with NT24 fermentation broth (0.5 μL/cm^2^) for 24 h exhibited more pronounced toxic symptoms, including higher degrees of paralysis and distortion (Figure 2A). Furthermore, the semi-lethal concentration (LC_50_) of NT24 was significantly reduced (Figure 2B). As shown, the LC_50_ values of the WT strain at 48 h were 1.890 μL/cm^2^, 1.262 μL/cm^2^ and 2.478 μL/cm^2^ against *H. armigera*, *S. exigua* and *S. litura*, respectively, while for NT24, the LC_50_ values were 1.305 μL/cm^2^, 0.511 μL/cm^2^ and 1.372 μL/cm^2^, demonstrating that the virulence of NT24 increased by 30.9%, 59.5% and 44.6%, respectively (Table 1).

### 2.3. Phenotypic Differences Between WT and NT24

**Figure 3:**
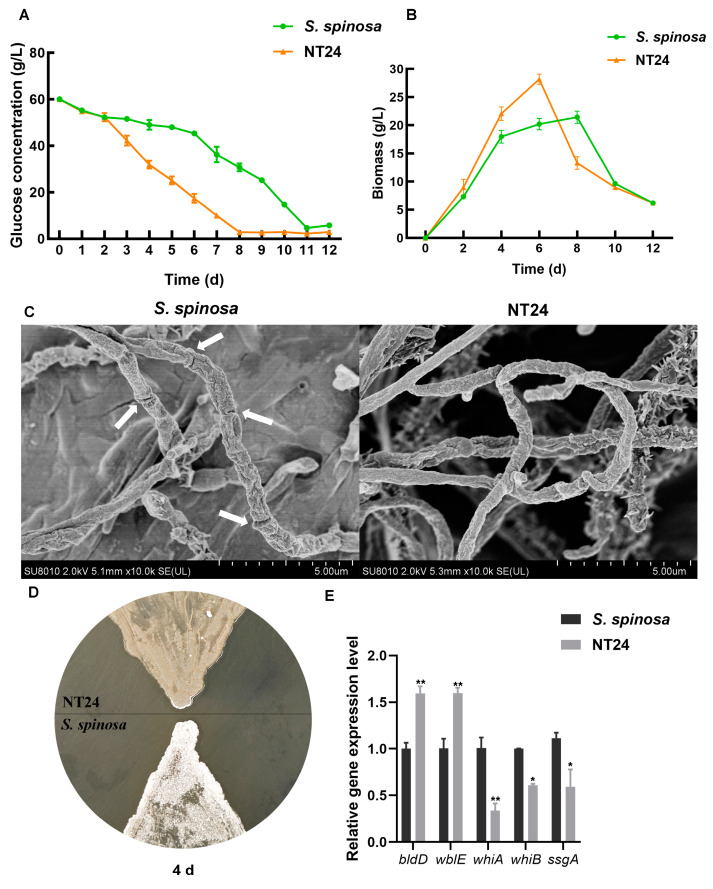
Effects of ARTP/NTG compound mutagenesis on strain growth and development. (**A**) Glucose consumption curve of WT and NT24. (**B**) Biomass accumulation curves of WT and NT24. (**C**) Mycelium comparison of WT and NT24 via SEM. The white arrows point to the differentiation nodes in WT. (**D**) Sporulation capacity comparison of WT and NT24. (**E**) Expression levels of genes related to sporulation and mycelium growth. Error bars are calculated from three independent determinations of mRNA abundance in each sample. Univariate variance, * *p* < 0.05, ** *p* < 0.005.

Given that the growth and metabolic capacity of strains often affect the biosynthesis of secondary metabolites, the determination of glucose consumption and biomass was employed to preliminarily assess the differences between the WT strain and NT24. The results indicate that the glucose consumption rate of NT24 exceeded that of WT from day 2, and its residual glucose level was only 2.8 g/L by day 8 (Figure 3A). The results of biomass determination show that the highest biomass of NT24 reached 29 g/L, which is significantly higher than that of WT, and the growth rate of NT24 was notably faster (Figure 3B). Furthermore, NT24 entered the decline phase on day 6, which was two days earlier than WT.

A comparison of spores and mycelium between the WT strain and NT24 was also conducted. The hyphal morphology during logarithmic growth (3 days) was observed via SEM. On day 3, the differentiation from mycelium to spores was observed in the WT strain, while the NT24 remained in the mycelial state (Figure 3C). The WT strain began to produce spores on day 4, while sporulation in NT24 was delayed until day 6 (Figure 3D). To elucidate this phenomenon, we determined the transcriptional levels of genes involved in mycelial development, including *bldD*, *wblE*, *ssgA*, *whiA*, and *whiB*. RT-qPCR (real-time quantitative polymerase chain reaction) analysis has shown that sporulation-related genes *bldD* and *wblE* were significantly upregulated, which may be the direct cause of the morphological differences between NT24 and the WT strain (Figure 3E).

### 2.4. Differences of the Accumulation in Spinosad and Short-Chain Acyl-coA

**Figure 4:**
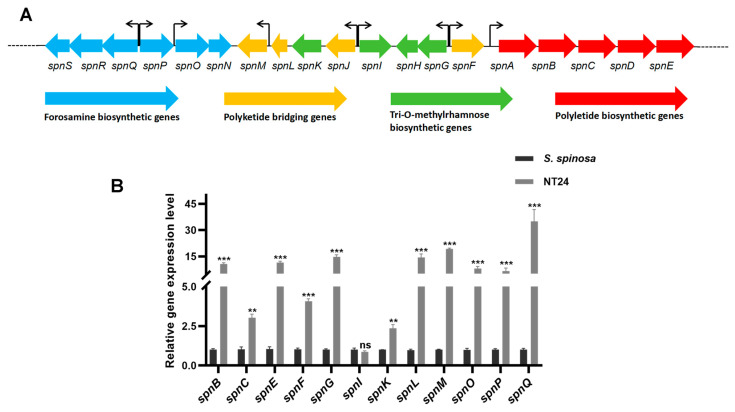
Transcription analysis of the spinosad biosynthetic gene cluster analyzed via RT-qPCR. (**A**) Main structure of the *spn* gene cluster and the transcription direction of each gene. (**B**) Transcript levels of representative *spn* genes. The error bars indicate the standard deviations of three biological replicates. Univariate variance, ^ns^ *p* > 0.05, ** *p* < 0.005, *** *p* < 0.001.

The spinosad gene cluster consists of 23 genes, 19 of which are *spn* genes (*spnA*-*spnS*) associated with forosamine biosynthesis, polyketide briding, tri-O-methylrhamnose biosynthesis and polyketide biosynthesis (Figure 4A). It is speculated that the increase in spinosad production in NT24 may be related to the activation of its spinosad gene cluster. Therefore, 12 *spn* genes were selected based on their transcription direction analyzed by RT-qPCR to evaluate the expression levels of the spinosad gene cluster (Figure 4B). It is obvious that almost all selected *spn* genes in NT24 were upregulated, suggesting that the increased spinosad production in NT24 is primarily mediated by the *spn* genes.

**Figure 5:**
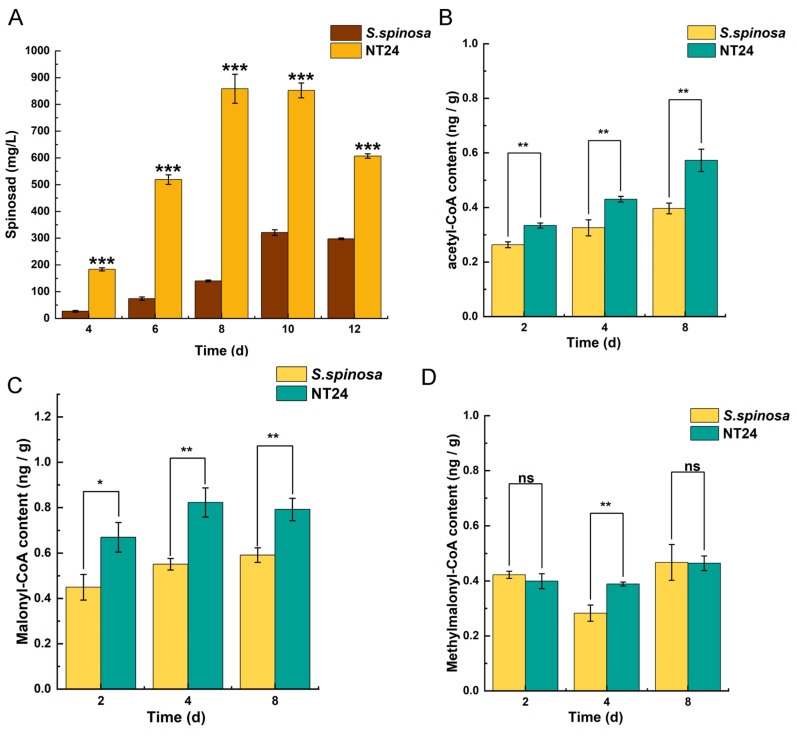
Effects of ARTP/NTG compound mutagenesis on the accumulation of spinosad and acyl-coA pool. (**A**) Spinosad accumulation of WT and NT24. (**B**) Acetyl-CoA accumulation of WT and NT24. (**C**) Malonyl-CoA accumulation of WT and NT24. (**D**) Methylmalonyl-CoA accumulation of WT and NT24. Univariate variance, ^ns^ *p* > 0.05, * *p* < 0.05, ** *p* < 0.005, *** *p* < 0.001.

Additionally, the supply of precursors plays a crucial role in the biosynthesis of spinosad. Therefore, the cumulative differences of spinosad production were analyzed in conjunction with the cumulative differences of its short-chain acyl-coA precursors. As shown, NT24 produced a substantial amount of spinosad by day 4 of fermentation, accumulating rapidly, with a maximum titer of 858.3 ± 27.7 mg/L on day 8, whereas the spinosad accumulation rate in WT was considerably slower (Figure 5A). The measurement of short-chain acyl-CoA precursors also revealed that the levels of acetyl-CoA and malonyl-CoA in NT24 increased significantly in all three periods (2d, 4d, 8d), and methylmalonyl-CoA increased significantly on day 4 (Figure 5B–D). These results indicate that the accumulation of acetyl-CoA and malonyl-CoA in NT24 influences its biosynthesis of spinosad. Moreover, malonyl-CoA and acetyl-CoA can be interconverted and stimulate acetyl-CoA synthesis, promoting the TCA cycle and thereby enhancing the growth activity of the strains.

### 2.5. Analyses of Resequencing and Metabolomics

Mutations are generally the primary cause of new phenotypes. Therefore, resequencing was performed on NT24 using the whole genome sequence of *S. spinosa* CCTCC M206084 as the reference. A total of 57 SNPs were identified in NT24, of which 48 were located in the CDS region and 30 caused non-synonymous mutations (Table S1). A total of 163 Indels were identified, 67 of which were located in the CDS, and 47 resulted in frameshift mutations (Table S2). The coding sequences of 49 genes were mutated, involving 30 enzymes, 13 proteins related to signaling and cellular processes, two regulators, one transposase and three hypothetical proteins. These mutations are enriched in signaling and cellular processes, carbohydrate metabolism, terpenoid and polyketides metabolism, amino acid metabolism, lipid metabolism and cell growth, purine and pyrimidine metabolism, pyruvate metabolism, and DNA replication and repair, according to the Kyoto Encyclopedia of Genes and Genomes (KEGG). Additionally, two SNPs and 50 Indels were found in the intergenic region of NT24.

Notably, SNPs were found in two genes directly related to pyruvate metabolism, *oadA* (oxaloacetate decarboxylase) and *poxB* (pyruvate oxidase). OadA catalyzes the irreversible decarboxylation of oxaloacetate to pyruvate, while PoxB is responsible for catalyzing the irreversible oxidation of pyruvate to acetate [38,39]. As an important intermediate metabolite, pyruvate can be converted into various precursors required for the biosynthesis of secondary metabolites, including spinosad. Therefore, we hypothesized that the mutations described above may lead to the accumulation of pyruvate in NT24, subsequently resulting in increased spinosad production.

**Figure 6:**
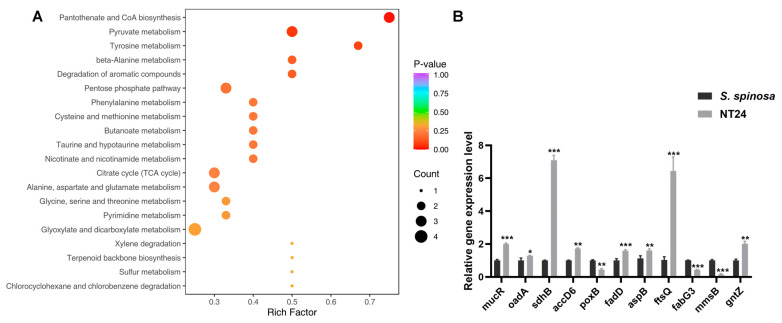
Representative differential genes and KEGG enrichment pathways in NT24. (**A**) Bubble plot of the most significant pathways with KEGG enrichment of DEGs. (**B**) Transcription levels of differential genes in NT24. Univariate variance, * *p* < 0.05, ** *p* < 0.005, *** *p* < 0.001.

Consequently, targeted metabolomics was employed to analyze differences in metabolite abundance associated with strain growth and target product biosynthesis by LC-MS/MS. The results show that among the 61 kinds of intracellular metabolites identified and quantified by LC-MS/MS, 34 exhibited significant differences, primarily including amino acids, carbohydrates, nucleotides and organic acids and their derivatives, with pyruvate abundance increasing significantly (Table S3). According to KEGG classification, the differential metabolites were mainly enriched in pantothenate and CoA biosynthesis, pyruvate metabolism, amino acid metabolism, the citrate cycle, and glyoxylate and dicarboxylate metabolism (Figure 6A).

RT-qPCR analysis of the mutated genes mentioned above was performed on WT and NT24 to verify the results of resequencing and metabolomics at the transcriptional level (Figure 6B). Differential gene analysis showed that the genes related with carbohydrate metabolism, such as *gntZ*, *sdhB*, *accD*, *oadA* and *fadD*, were upregulated, while *mmsB* and *poxB* were downregulated. In addition, the relative transcription levels of *aspB* (coding aspartate aminotransferase) and *ftsQ* (cell division protein FtsQ) were downregulated.

## 3. Discussion

**Figure 7:**
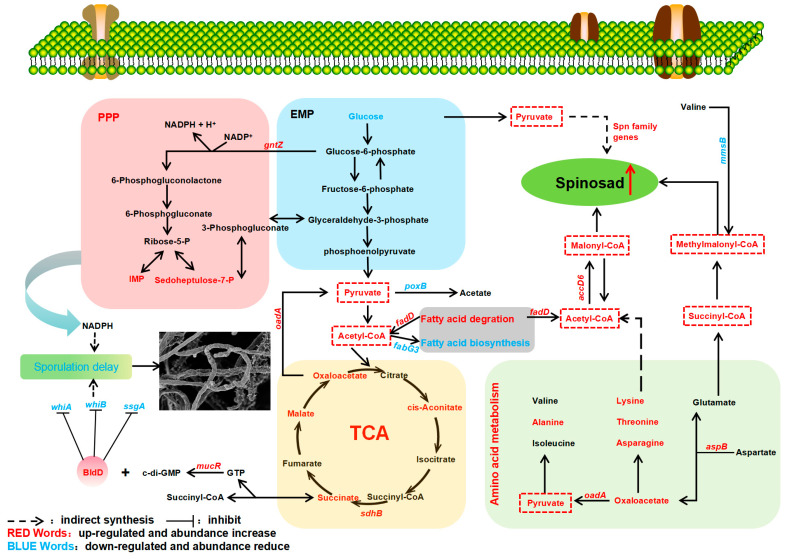
Effects of ARTP/NTG compound mutagenesis on primary and secondary metabolisms of NT24. The pyruvate and acyl-CoA pools are marked with a red box.

Natural products are the result of a long-term evolutionary process of microbial adaptation to the environment, and are closely related to a strain’s growth and metabolism [40]. Exploring key genes and their effects on strain growth and secondary metabolism will help to reveal the regulatory mechanism of spinosad biosynthesis and provide guidance for the optimization of other polyketides’ production (Figure 7). As shown by biomass measurements, the biomass of NT24 was much higher than that of WT, which may be one of the reasons for the increased yield of spinosad in NT24. *ftsQ* is one of the differential genes in NT24. At the end of a highly conserved cell wall division operon, the *ftsQ* gene encodes a cell division protein that plays an important role in regulating cell growth and death and maintaining cell length, which is necessary for chromosome separation and eventual cell division [41]. The increasing biomass in NT24 may be related to the upregulation of *ftsQ*, which indirectly leads to the increase in spinosad. As for the morphological observation, we found that the sporulation of NT24 was delayed, and SEM observation showed more clearly that the mycelium of NT24 did not show differentiation at day 3. Therefore, the genes related to sporulation and mycelial growth were verified by RT-qPCR, among which the global regulator *bldD* was upregulated 1.6-fold. BldD usually acts as a developmental repressor to control the morphological development of *Streptomyces*, and can also directly regulate the biosynthesis of secondary metabolites [42,43,44]. In addition, we found an SNP in the promoter region of *mucR*, the diguanylate cyclase coding gene, and the transcriptional level of *mucR* in NT24 was also upregulated. According to previous studies, mutations in a limited number of bases in a promoter can induce significant changes in protein expression [39,45]. Diguanylate cyclase catalyzes GTP cyclization to form c-di-GMP, a signaling molecule with diverse functions, which can regulate the activity of *bldD* and bind with *bldD* to cope with stress or nutrient starvation, and inhibit the differentiation from mycelium to spore as well [46]. Furthermore, c-di-GMP has been verified to participate in the cascade regulation of secondary metabolite biosynthesis [47,48], but its regulation of spinosad biosynthesis remains to be further studied.

The results of spinosad accumulation show that the fermentation cycle of NT24 was shortened by two days, reaching the highest titer of 858.3 ± 27.7 mg/L on the 8th day, which is consistent with the growth trend. The glucose consumption assay showed an accelerated rate of glucose consumption in NT24, which may be related to the previously mentioned acceleration of cell division. Accelerated cell division resulted in more cells in a short period, thus consuming more glucose. In addition, the RT-qPCR results show that the 6-phosphogluconate dehydrogenase (an important enzyme in the pentose phosphate pathway, PPP) coding gene *gntZ* was significantly upregulated in NT24, which catalyzes the generation of ribulose from glucose-6-phosphate. Ribulose can further be converted into sedoheptulose-7-phosphate or IMP. Targeted metabolomics also confirmed significantly increased levels of sedoheptulose-7-phosphate and IMP, suggesting that the upregulation of *gntZ* led to an increase in PPP pathway flux. Meanwhile, PPP is an important source of NADPH, and according to recent analyses of biological intracellular metabolite levels, the balance between NADH production and NADPH consumption by redox metabolism affects the biosynthesis and morphological differentiation of secondary metabolites [49,50].

Spn family genes are key regulatory genes of spinosad biosynthesis, directly affecting the efficiency of its production. The RT-qPCR results showed that almost all selected *spn* genes were upregulated in NT24, which is the direct reason for the improved spinosad production. Moreover, the accumulation of short-chain acyl-CoA precursors is also an important reason for the increase in spinosad. Targeted metabolomics showed that pyruvate content in NT24 increased significantly, and the conversion of pyruvate could alter the contents of propionyl-CoA, malonyl-CoA and methylmalonyl-CoA, ultimately affecting spinosad biosynthesis [51,52]. Therefore, we conclude that pyruvate accumulation indirectly improved the spinosad biosynthesis pathway. Combined with the transcriptional-level analysis of differential genes identified in resequencing and key genes in corresponding metabolic pathways, we found a large metabolic flow into pyruvate and short-chain acyl-coA precursors. RT-qPCR showed that *aspB*, *sdhB*, *accD6*, and *fadD* were significantly upregulated, while *poxB*, *fabG3*, and *mmsB* were significantly downregulated. Aspartate and alpha-ketoglutaric acid can be catalyzed by aspartate aminotransferase (*aspB*) to form oxaloacetate and glutamic acid. Then, oxaloacetic acid can be converted to pyruvate under the catalysis of oxaloacetate decarboxylase (*oadA*) in the glyoxylate cycle, and glutamic acid can be converted to succinyl-CoA, the precursor of spinosad biosynthesis. In NT24, the abundances of malate, succinate, oxaloacetate, and cis-aconitate in the TCA cycle were significantly increased, and the succinate dehydrogenase coding gene *sdhB* was significantly upregulated, indicating the enhancement of the TCA cycle, which might be one of the reasons for the enhanced growth activity of NT24. Long-chain acyl-CoA synthetase (*fadD*) and short-chain dehydrogenase (*fabG3*) are two important rate-limiting enzymes in fatty acid degradation and biosynthesis, respectively. The upregulation of *fadD* and downregulation of *fabG3* enhance fatty acid degradation, which produces a large amount of acetyl-CoA, reducing the competition for acetyl-CoA in fatty acid biosynthesis. In addition, acetyl-CoA can be catalyzed by acetyl-CoA carboxylase (*accD6*) and converted into malonyl-CoA, the direct precursor of spinosad biosynthesis, which is consistent with the above results. The downregulation of *mmsB* (encoding 3-hydroxyisobutyrate dehydrogenase) partly explains why the increasing level of methylmalonyl-CoA was not as high as the levels of acetyl-CoA and malonyl-CoA.

## 4. Materials and Methods

### 4.1. Strains and Culture Conditions

The wild-type strain *S. spinosa* CCTCC M206084 (WT, GenBank accession no. CP061007) used in this study was stored in our lab. All the strains used in this study were listed in Table S4. The CSM medium (10 g/L glucose, 45 g/L trypticase soy broth, 9 g/L yeast extract, and 2.2 g/L MgSO_4_·7H_2_O) was used for the seed culture in a 100 mL flask, with a starting volume of 20 mL at 30 °C and 260 rpm. After 48 h of cultivation, 1.5 mL activated bacterial solution was inoculated into 30 mL fermentation media (60 g/L glucose; 22.5 g/L cottonseed cake meal, 5 g/L bean cake powder, 2 g/L yeast extract, 10 g/L soluble starch, 7 g/L corn syrup, 5 g/L CaCO_3_, pH = 7.5) and incubated at 30 °C at 260 rpm, for 10–12 days. All the media were autoclaved at 115 °C for 30 min to confirm the sterility of each batch of broth. Spore morphology was observed in TSB agar plates (tryptic soy broth medium) and incubated at 30 °C for 6 days. All reagents were purchased from Tianheng Biotechnology Co. LTD (Beijing, China).

### 4.2. Semi-Lethal Concentration Assay of WT and Mutant Strain NT24

The larvae (KEYUN Biology Technology Inc) of *Helicoverpa armigera* (*H. armigera*), *Spodoptera exigua* (*S. exigua*) and *Spodoptera litura* (*S. litura*) were fed with artificial feed at 28 °C with a light/dark cycle of 12 h. Each well of the 24-well cell culture plates (Corning, USA) was added with 0.25 cm^3^ (1 cm × 1 cm × 0.25 cm) artificial feed. After the WT and NT24 were fermented for 12 days, the fermentation broth was collected and the artificial feed was sprayed with 0.5, 1, 1.5, 2 and 2.5 (μL/cm^2^) of fermentation broth, ensuring full coverage of the feed surface. The blank control was the corresponding concentration of normal saline. The larvae were cultured in the 24-well cell culture plates (one larva per well) with three replicates. The survival percentage of the larva was recorded after 48 h.

### 4.3. Detection and Identification of Spinosad

Under the corresponding fermentation time, 3 mL methyl alcohol was added to 1 mL fermentation broth, and the supernatant was centrifuged at 12,000× *g* rpm for 10 min for high-performance liquid chromatography and LC-MS/MS analysis. HPLC (Agilent 1290, wavelength: 250 nm, C18 column: AQ12S05-1546WT, YMC) was used to detect the spinosad titer. Here, 20 μL sample was loaded onto a C18 column (4.5 μm, 4.5 × 150 mm, AQ12S05-1546WT) and detected at 250 nm for 25 min with a rate of 1 mL/min. The mobile phase consisted of acetonitrile (42%), methyl alcohol (42%) and 20 g/L ammonium acetate (16%). The spinosad peak was identified by LC-MS/MS on the LTQ-XL (Thermo Fisher, Waltham, MA, USA) as in the previous study [53].

### 4.4. ARTP/NTG Mutagenesis

ARTP mutagenesis was performed in the ARTP biological mutagenesis system (Wuxi Yuanqing Tianmu Biological Technology Co., Ltd., Wuxi, China), with a 100 W working radio-frequency power input, 2.0 mm treatment distance and 10 standard liters per minute (SLPM) gas flow. To determine the optimal treatment time, the WT strain’s spore suspension (107–108 CFU/mL) was treated in a range of 0–150 s, and the untreated spore suspension was used as control. The treatment time that resulted in a 90% mortality of the strain was selected as the optimal treat duration. The resuspend cell broth was diluted, spread onto TSB agar plates and incubated in 30 °C for 6 days. Each clone on these plates was activated on 2 mL CSM medium for 48 h, and a sample from 3 random sights was selected to observe the mycelium; the mutants containing stronger mycelium were screened out and cultured in the fermentation media at 30 °C for 12 days.

HPLC was used to detect the spinosad titer. The mutant strain with the highest spinosad titer in ARTP mutagenesis was used as the original strain in NTG mutagenesis. And the optimum NTG concentration was selected from the lethality rates (reach 90%) under different NTG concentrations (0, 100, 200, 300, 400, and 500 µg/mL) for 1 h. The follow-up steps here were the same as in ARTP mutagenesis.

### 4.5. Growth Curve and Glucose Consumption Determination

During fermentation, 5 mL of fermentation broth was collected from the shake flask every 2 days, filtered through three layers of pre-weighed filter paper, and placed in a 50 °C oven. After 12 h, the sample was removed and re-weighed. The growth curve was measured over 12 days with three biological replicates. To determine the glucose content, 1.5 mL dinitrosalicylic acid reagent was added to 1 mL of fermentation broth supernatant daily during the 12-day fermentation with three biological replicates. The mixture was incubated in a water bath at 100 °C for 2 min. After adding ultrapure water to a final volume of 10 mL, the absorbance was measured (540 nm), and glucose content was calculated based on the dilution factor and optical density (OD) value.

### 4.6. Morphological Observation

The mutants activated in CSM medium for 48 h were observed with a phase contrast microscope (AXIO Scope A1, Zeiss, Germany). WT and NT24 were activated in CSM medium for 48 h, and 20 μL of each was taken out and spread on TSB solid medium. After that, the sterilized cover glass was inserted into TSB solid medium aslant. After 3 days of culture at 30 °C, the cover glass was taken out and dried for sputtering gold plating, and then imaged with a scanning electron microscope (SEM, Hitachi, Shinagawa-ku SU8010, Japan).

### 4.7. Determination of Short-Chain Acyl-CoA Precursors

Biomass samples of WT an NT24 were harvested on the 2nd, 4th, and 8th days, and sufficiently broken by grinding with liquid nitrogen to release intracellular components. The supernatants were collected (8000 rpm, 10 min) and analyzed according to the manufacturers’ instructions using microorganism acetyl-CoA/malonyl-CoA/methyl-malonyl-CoA ELISA Kits (Jiangsu JINMEI Biotechnology Co., Ltd., Jiangsu, China). Three biological replicates were conducted in this experiment.

### 4.8. Genome Sequencing and Assembly

The genome of NT24 was sequenced using an DNBSEQ platform at the Beijing Genomics Institute (Shenzhen, China). Genomic DNA was sheared randomly to construct three read libraries with lengths of 8738,130 bp via physico-chemical methods. The paired-end fragment libraries were sequenced. Raw reads of low quality produced by paired-end sequencing (those with consecutive bases covered by fewer than five reads) were discarded. The sequenced reads were assembled using SOAPdenovo v1.05 software. All mentioned metabolites were detected by MetWare (http://www.metware.cn/) (accessed on 2 February 2024) based on the AB Sciex QTRAP 6500 LC-MS/MS platform.

### 4.9. Total RNA Extraction and RT-qPCR Analysis

The total RNA of WT and NT24 at 96 h was extracted following the instructions for the total RNA Extractor (Sangon, Shanghai, China). NanoDrop 2000 (Thermo) was used to test the purity and concentration of extracted RNA. The total RNA was reverse-transcribed into cDNA using a RevertAidTM First Strand cDNA Synthesis Kit (Fermentas, Waltham, MA, USA) following the instructions. All samples were analyzed on a 7500 Real-Time PCR system instruments (Applied Biosystems, Norwalk, CT, USA). The 16S rRNA gene was regarded as the endogenous control to quantify the relative expression levels of the target genes. The RT-qPCR primers used in this study are listed in Table S5. The experiment was repeated 3 times.

### 4.10. Stability Analysis

Genetic stability testing was conducted by subculturing NT24 in 30 mL of fermentation medium for five passages (the transduction was performed once every 8 days), and each passage was subjected to HPLC analysis.

### 4.11. Statistic Method

The mortality of larvae (%) = (A/72) × 100%, where A is the number of dead larvae counted across three 24-well plates. The LC_50_ and statistical significance analysis were performed using the SPSS software (Inc, version 20, Chicago, IL, USA). The lethality rate of ARTP/NTG mutagenesis (%) = ((A − B)/A) × 100%, where A is the number of control colonies on TSB plate, and B is the number of ARTP/NTG mutagenesis colonies at the same gradient.

## 5. Conclusions

In conclusion, a mutant strain NT24 with enhanced spinosad biosynthesis was obtained through ARTP/NTG compound mutagenesis in this study, where the accumulation of pyruvate and acyl-CoA precursors plays a central role in spinosad production. The four key genes (*oadA*, *poxB*, *accD6* and *fadD*), identified through metabolomics and RT-qPCR, could be overexpressed or knocked out/down in future studies to validate their functions and effects on spinosad biosynthesis. In addition, in contrast to genetically engineered strains (mutant strains obtained through genetic modification), the mutant strains obtained via ARTP/NTG compound mutagenesis circumvent the regulatory restrictions on genetically modified products in agricultural applications, offering greater versatility. Moreover, the mutant strain generated from mutagenesis can serve as an “original strain” for further genetic modifications to enhance its spinosad titer. The method adopted in this study can also be applied to optimize the biosynthesis of secondary metabolites in other actinomycetes.

## Figures and Tables

**Table 1:** LC_50_ analysis of the toxicity of WT and NT24 against *H. armigera*, *S. exigua*, and *S. litura*.

Strains	LC_50_ (μL/cm^2^)			95% Confidence Interval		
	Pest					
	*H. armigera*	*S. exigua*	*S. litura*	*H. armigera*	*S. exigua*	*S. litura*
*S. spinosa*	1.890	1.262	2.478	1.631–2.259	1.057–1.439	2.176–3.028
NT24	1.305	0.511	1.372	1.053–1.521	0.092–0.768	1.195–1.539

## Data Availability

The raw data supporting the conclusions of this article will be made available by the authors on request.

## Supplementary Materials

The following supporting information can be downloaded at https://www.mdpi.com/article/10.3390/ijms252212308/s1.
