# Supplementary material for: ARTP/NTG Compound Mutagenesis Improved the Spinosad Production and the Insecticidal Virulence of Saccharopolyspora Spinosa
Source: Int J Mol Sci. 2024 Nov 16;25(22):12308. doi: 10.3390/ijms252212308 (PMC11594378; doi:10.3390/ijms252212308)
Supplement: Supplementary file 1 [file ijms-25-12308-s001.zip › ijms-3263995-supplementary.pdf]

Title:

ARTP/NTG compound mutagenesis improved the spinosad production and the insecti-cidal virulence of *Saccharopolyspora spinosa*

Zirong Zhu[#], Wangqiong chen[#], Li Cao, Ziyuan Xia, Jie Rang, Shengbiao Hu, and Liqiu Xia[*]

Hunan Provincial Key Laboratory of Microbial Molecular Biology, State Key Laboratory of Developmental Biology of Freshwater Fish, College of Life Science, Hunan Normal University, Changsha 410081, Hunan, China.

[*]Corresponding author:

E-mail: xialq@hunnu.edu.cn

Tel/ Fax: +86 73188872298.

[#]These authors contributed equally to this study

Figures legends

Figure S1. The mass spectrometry identification of the fermentation products in NT24.

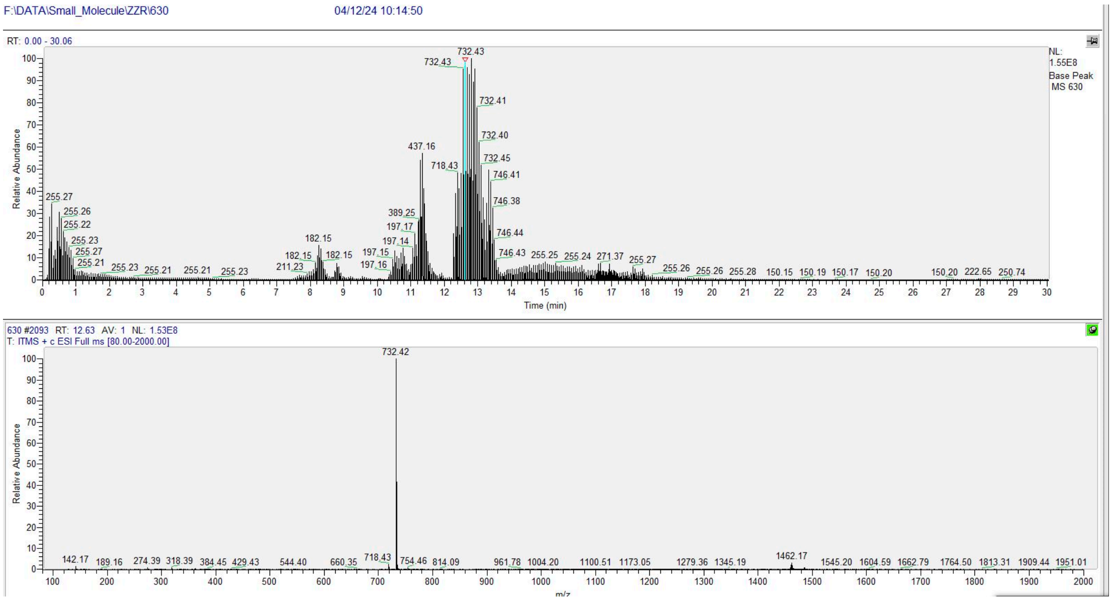

Tables

Table S1 SNP sites in CDS.

Position	Base mutate	AA mutate	Mutate type	Gene id
4936	G<->A	D<->N	Nonsynonymous	S360GL000004
6739	G<->A	G<->G	Synonymous	S360GL000006
7996	G<->A	A<->T	Nonsynonymous	S360GL000007
13113	G<->A	A<->T	Nonsynonymous	S360GL000011
26710	G<->A	A<->T	Nonsynonymous	S360GL000030
31033	C<->T	A<->V	Nonsynonymous	S360GL000033
85937	G<->A	D<->N	Nonsynonymous	S360GL000089
94319	G<->A	A<->A	Synonymous	S360GL000100
187209	C<->T	L<->F	Nonsynonymous	S360GL000196
228040	G<->A	A<->T	Nonsynonymous	S360GL000244
711495	C<->T	D<->D	Synonymous	S360GL000758
795554	C<->T	D<->D	Synonymous	S360GL000856
1105440	C<->T	P<->L	Nonsynonymous	S360GL001171
1193444	G<->A	A<->T	Nonsynonymous	S360GL001262
1245657	C<->T	S<->S	Synonymous	S360GL001326
1287494	G<->A	E<->E	Synonymous	S360GL001369
1346803	C<->T	N<->N	Synonymous	S360GL001425
1512116	G<->A	G<->D	Nonsynonymous	S360GL001588
2191381	C<->T	T<->I	Nonsynonymous	S360GL002335
2324511	C<->T	L<->F	Nonsynonymous	S360GL002478
2459887	G<->A	Q<->Q	Synonymous	S360GL002608
3021020	C<->T	G<->G	Synonymous	S360GL003184
3110590	G<->A	G<->D	Nonsynonymous	S360GL003280
3506842	C<->T	S<->S	Synonymous	S360GL003650
4131004	A<->G	T<->A	Nonsynonymous	S360GL004308
4144463	G<->A	E<->K	Nonsynonymous	S360GL004321
4381379	C<->T	L<->L	Synonymous	S360GL004581
4494224	C<->T	P<->S	Nonsynonymous	S360GL004720
5026809	C<->T	A<->A	Synonymous	S360GL005291
5119515	G<->A	Q<->Q	Synonymous	S360GL005408
5198728	G<->A	A<->T	Nonsynonymous	S360GL005496
5280520	C<->T	Q<->*	Premature_stop	S360GL005583
5498521	G<->T	G<->V	Nonsynonymous	S360GL005761
5862070	G<->A	W<->*	Premature_stop	S360GL006036
5911178	C<->T	H<->H	Synonymous	S360GL006089
6194297	C<->T	A<->V	Nonsynonymous	S360GL006317
6284944	C<->T	P<->L	Nonsynonymous	S360GL006398
6312795	G<->A	G<->G	Synonymous	S360GL006425
6370118	T<->C	I<->T	Nonsynonymous	S360GL006469
6681480	G<->A	R<->Q	Nonsynonymous	S360GL006778
7704248	A<->G	S<->G	Nonsynonymous	S360GL007831
7741903	C<->T	P<->L	Nonsynonymous	S360GL007864
8099311	T<->C	C<->R	Nonsynonymous	S360GL008256
8130970	C<->T	S<->S	Synonymous	S360GL008297
8384414	G<->A	E<->K	Nonsynonymous	S360GL008600
8495450	T<->C	L<->P	Nonsynonymous	S360GL008692
8697628	G<->A	R<->Q	Nonsynonymous	S360GL008929
8707855	A<->G	D<->G	Nonsynonymous	S360GL008941

Table S2 Indel sites in CDS.

Position	InDel_type	InDel_start	InDel_end	Base	Gene id
7022266	I2	7021998	7022423	CG	S360GL007105
6601642	I5	6601597	6602043	CCGGG	S360GL006688
8769853	I6	8769420	8770223	CCGGGC	S360GL009006
6281581	I6	6279837	6291200	GCCGGC	S360GL006398
6096942	I5	6096926	6097180	CCGGC	S360GL006223
2262945	I3	2262930	2263409	CCG	S360GL002410
6438428	I4	6437822	6438583	CCGG	S360GL006536
3800474	D2	3800396	3800704	TA	S360GL003944
151787	I2	151591	154347	TG	S360GL000159
1703505	I5	1702762	1703574	GGCCG	S360GL001806
2723065	I1	2723017	2723820	G	S360GL002931
7423804	I1	7423748	7424401	G	S360GL007534
7399189	I5	7398402	7399649	CCGGC	S360GL007508
2026448	I5	2026445	2027236	CCGAG	S360GL002151
5041381	I1	5041244	5041426	C	S360GL005307
6578283	I1	6578272	6578859	C	S360GL006676
4351573	I5	4351109	4351759	CCGGC	S360GL004550
5187939	I5	5187630	5188076	GCAGC	S360GL005481
4520206	I6	4519346	4522042	CCGGCC	S360GL004754
5947603	I1	5946836	5947618	C	S360GL006124
3952157	I5	3951767	3952720	GAGCC	S360GL004105
5962648	I3	5962591	5962713	GCT	S360GL006140
3026639	I6	3026448	3026705	AGCCGG	S360GL003190
182666	I5	182500	182892	AAGCC	S360GL000187
4287660	I5	4287658	4288620	CCGGC	S360GL004485
7168415	I6	7167235	7169604	CGCCGG	S360GL007256
6694889	I3	6693654	6694889	GCC	S360GL006789
5534406	I2	5534139	5535515	GC	S360GL005789
8296163	I9	8295979	8297586	CGTTGTAGC	S360GL008493
7643093	I5	7642927	7643835	GCCGG	S360GL007770
639648	I1	639615	641267	C	S360GL000672
3229624	I1	3227661	3229862	G	S360GL003391
8125200	I6	8125028	8125249	TGGGCC	S360GL008288
2148716	I3	2148403	2148717	CCG	S360GL002288
3239444	I1	3239269	3239508	G	S360GL003399
5245577	I1	5244685	5247354	T	S360GL005546
4083555	I6	4083523	4083879	CCGGCT	S360GL004252
608667	D2	608518	609435	TA	S360GL000641
3751679	I3	3751603	3752415	CGG	S360GL003889
6219189	I1	6219164	6219364	C	S360GL006339
6025805	I7	6025627	6039450	CGCCGGC	S360GL006198
341489	I1	341066	343444	C	S360GL000361
4428541	I6	4428227	4428568	GGCTCG	S360GL004644
7013449	I3	7012939	7013517	CGG	S360GL007093
3447701	I6	3447611	3448645	CACAAC	S360GL003588
3535723	I1	3535712	3536593	G	S360GL003677
1548487	I5	1548049	1548687	GCCGG	S360GL001622
4938842	I6	4938443	4939321	CGGCCG	S360GL005197
111817	I1	111788	111907	T	S360GL000118
4131003	I2	4130849	4131013	GC	S360GL004308
4259833	I6	4259359	4260009	CCGGCC	S360GL004453
3688042	I4	3687921	3688100	CGGC	S360GL003819

8449618	I1	8449394	8450287	C	S360GL008653
6478982	I1	6478935	6482648	C	S360GL006586
8938912	I6	8938244	8939089	GGCCGG	S360GL009196
5823692	I5	5823544	5824191	GCCGG	S360GL005994
8020936	I2	8020522	8020983	CT	S360GL008165
6483185	I2	6483171	6484355	CC	S360GL006588
7612276	I5	7612269	7612913	CCGGC	S360GL007738
8695024	I5	8692811	8696257	CCGGC	S360GL008928
2555016	I5	2554945	2555352	GCCGG	S360GL002722
5227193	I5	5227189	5227353	GCCGG	S360GL005528
560551	I3	560515	560814	CCG	S360GL000581
6078353	I3	6078316	6079041	CCG	S360GL006203
6578283	I1	6575519	6578290	C	S360GL006675
5498482	D1	5498412	5500352	C	S360GL005761
6477254	I1	6477207	6478718	C	S360GL006584

Table S3 Differential expression of metabolites related to energy metabolism in NT24.

Compounds	Class	Fold_Change	Pvalue	Type
L-Asparagine	Amino acids	1.44	$4.28e^{-4}$	up
L-Alanine		1.47	$8.19e^{-4}$	up
L-Leucine		1.36	$2.93e^{-5}$	up
L-citrulline		1.66	$3.88e^{-5}$	up
Arginine		1.47	0.001	up
Tyrosine		1.72	0.008	up
Lysine		1.24	0.012	up
Serine		1.41	0.007	up
Threonine		1.34	0.001	up
DL-Glyceric-Acid	Carbohydrate metabolomics	4.12	0.072	up
Ureidopropionate		2.68	0.005	up
Gluconate		0.48	$9.94e^{-4}$	down
Cysteic-acid		3.42	$3.60e^{-4}$	up
D(+)-Glucose		0.61	0.004	down
Flavin-mononucleotide	CoEnzyme and vitamins	1.67	0.017	up
Succinyl-CoA		1.30	0.028	up
Guanosine	Nucleotide and Its metabolomics	0.5	0.001	down
UMP		1.70	0.003	up
AMP		1.39	0.044	up
dTMP		1.43	0.017	up
Uracil		3.61	0.004	up
IMP		1.59	0.022	up
NADPH		0.47	0.110	down
Adenine		2.01	0.006	up
Malic-acid	Organic acid And Its derivatives	2.68	0.001	up
Oxaloacetate		1.51	0.001	up
cis-Aconitic-acid		1.39	0.033	up
Argininosuccinic-acid		2.23	0.033	up
Pyruvic acid		4.27	0.019	up

Succinic Acid		2.72	2.15e^{-4}	up
3-phenyllactic acid		1.73	0.002	up
Trehalose-6-phosphate		1.52	0.037	up
Sedoheptulose-7-phosphate	Phosphate sugars	1.50	0.047	up
Glycerol-3-phosphate		0.60	0.005	down

Table S4 Strains used in this study

	Relative description	Sources
**Strains**		
S. spinosa	The wild strain used as starting strain	Lab store
A5	ARTP mutation from *S. spinosa*	This work
NT24	ARTP/NTG compound mutation from A5 (the highest titer)	This work
NT42	ARTP/NTG compound mutation from A5	This work
NT60	ARTP/NTG compound mutation from A5	This work

Table S5 qRT-PCR Primers used in this study

Primers	Sequence (5′→ 3′)	Sources
qR-*16S*	CGTCAGCTCGTGTCGTGAGA	This work
qR-*16S*	GTGAAGCCCTGGGCATAAGG	This work
qF-*spnB*	TACGGGAGCGAAACAAATC	This work
qR-*spnB*	ACCACAGCACCCAACAGC	This work

qF-*spnC*	GGATGTGGTGATGGGGCA	This work
qR-*spnC*	CGTCAGAGGGCAGGGCTT	This work
qF-*spnE*	CATTGACCCGTTTTCTTTGC	This work
qR-*spnE*	CCTCTAACCCGAAGGTGTAAGC	This work
qF-*spnF*	CAGGTTGGGCAGATGTATGAC	This work
qR-*spnF*	GGCGACAAGGTCGGTGAG	This work
qF-*spnG*	CCGCAGTGTTCTCCAGTGTC	This work
qR-*spnG*	CCAGTCCGACCACGAACAGT	This work
qF-*spnO*	CGACGGTAGGTTCTTCACGG	This work
qR-*spnO*	TGACGAGAATGCCGAGGATAC	This work
qF-*spnI*	GCGAGGTGACCAGAGCGAT	This work
qR-*spnI*	GGAAACAGGGATTGGAAGGATT	This work
qF-*spnK*	GCGATGTTGTGGTAGGTGTGG	This work
qR-*spnK*	CACGGTCAGCCTGCTCAAG	This work
qF-*spnL*	TGCTTTCCTCGTTTCTGGC	This work
qR-*spnL*	CAGCAACGCTACTTGGGAG	This work
qF-*spnM*	GACGGTGTAGGTGTGGGTTC	This work
qR-*spnM*	GTAGCCAAGTTGCCCCG	This work

qF-*spnP*	GATGCGTTTCGTGCCGTA	This work
qR-*spnP*	TACTGTTCGGTCACCCTGC	This work
qF-*spnQ*	TCTGGCGTTGCGTTGTAGG	This work
qR-*spnQ*	CGCTTACCTCCCCCAAACT	This work
qF-*bldD*	CTATCCGCCAGCAGCAGG	This work
qR-*bldD*	ATAGGACCCGACGACCAC	This work
qF-*wblE*	AGTGGTCCTGCTGTGCTGC	This work
qR-*wblE*	CCGCTCGTCTTCGCTCAT	This work
qF-*whiA*	CCGACGGGCTGAGGTTTC	This work
qR-*whiA*	GTGCCCGAACAGCTCGTG	This work
qF-*whiB*	GAGGCGACGGACGAAGAAC	This work
qR-*whiB*	GCGTTCCCTTTCGGACAGT	This work
qF-*ssgA*	GTTGGCTCCGCAGACACCG	This work
qR-*ssgA*	AGACCGTCGGCGAGCAGG	This work
qF-*mucR*	AAGCGGACCGACAGCCAT	This work
qR-*mucR*	GTGGGTAGCCTGATAGTGGAGTT	This work
qF-*mmsB*	CCGCAACACGGCTGAAAC	This work
qR-*mmsB*	GCTGAGCCCTTGAACTGCG	This work

qF-*gntZ*	GTCCGCTCCTGGCTGCT	This work
qR-*gntZ*	GCGTTGTTGATCGATTCCTC	This work
qF-*ftsQ*	GACGCTCGCCCAGGAACA	This work
qR-*ftsQ*	AGCTCAGCGACACCTGCG	This work
qF-*poxB*	GAGTGGGTCCACGTGCGC	This work
qR-*poxB*	CACCAGCACGGAAACGCC	This work
qF-*oadA*	CACACCGTTCTCCCAGTTCG	This work
qR-*oadA*	CCGTAGTGACCAAGGGCGTA	This work
qF-*sdhB*	CAAAGACAACGAGCAGGCG	This work
qR-*sdhB*	TGATGTTGCACAGGCCCA	This work
qF-*accD6*	GTGGAGTCGCTGGATGGC	This work
qR-*accD6*	GATCACCACATCGGTCAACG	This work
qF-*aspB*	GAACCCACCTACTCCCTCTACC	This work
qR-*aspB*	GGTTGCACAGCACGATCATC	This work
qF-*fadD*	GAGGTCGGCACCATCTACTTC	This work
qR-*fadD*	CATGGTTGCCCAGTTCTCG	This work
qF-*fabG3*	GACCCGACGCTGCAACAC	This work
qR-*fabG3*	GCACACCTCCTCCACCACT	This work
